# The Relationship between Initial Tacrolimus Metabolism Rate and Recipients Body Composition in Kidney Transplantation

**DOI:** 10.3390/jcm10245793

**Published:** 2021-12-10

**Authors:** Aureliusz Kolonko, Patrycja Pokora, Natalia Słabiak-Błaż, Beata Czerwieńska, Henryk Karkoszka, Piotr Kuczera, Grzegorz Piecha, Andrzej Więcek

**Affiliations:** Department of Nephrology, Transplantation and Internal Medicine, Medical University of Silesia, Francuska 20/24, 40-027 Katowice, Poland; pat.pokora@gmail.com (P.P.); nataliablaz@gazeta.pl (N.S.-B.); bczerwienska@op.pl (B.C.); hkarkoszka@poczta.fm (H.K.); p.m.kuczera@gmail.com (P.K.); g.piecha@outlook.com (G.P.); awiecek@sum.edu.pl (A.W.)

**Keywords:** bioimpedance analysis, drug dosing, lean body mass index, pharmacokinetics, tacrolimus C/D ratio

## Abstract

There are several premises that the body composition of kidney transplant recipients may play a role in tacrolimus metabolism early after transplantation. The present study aimed at analyzing the relationship between the body composition parameters assessed by bioimpedance analysis (BIA) and initial tacrolimus metabolism. Immediately prior to transplantation, BIA using InBody 770 device was performed in 122 subjects. Tacrolimus concentration-to-dose (C/D) ratio was calculated based on the first blood trough level measurement. There was no difference in phase angle, visceral fat area, lean body mass index (LBMI) and the proportion of lean mass as a percentage of total body mass between the subgroups of slow and fast metabolizers. However, subjects with LBMI ≥ median value of 18.7 kg/m^2^, despite similar initial tacrolimus dose per kg of body weight, were characterized by a significantly lower tacrolimus C/D ratio (median 1.39 vs. 1.67, respectively; *p* < 0.05) in comparison with the subgroup of lower LBMI. Multivariate regression analysis confirmed that age (r_partial_ = 0.322; *p* < 0.001) and LBMI (r_partial_ = −0.254; *p* < 0.01) independently influenced the tacrolimus C/D ratio. A LBMI assessed by BIA may influence the tacrolimus metabolism in the early post-transplant period and can be a useful in the optimization of initial tacrolimus dosing.

## 1. Introduction

Nowadays, the calcineurin inhibitor tacrolimus is the primary therapeutic option for patients after kidney transplantation. However, due to its substantial intra- and inter-patient variability in metabolism rate the accurate dosing is still challenging [1,2,3]. Moreover, many external factors also influence the tacrolimus blood trough level, making the frequent drug therapeutic monitoring mandatory [4,5,6]. As an inappropriately high or low tacrolimus level in the early post-transplant period can result in delayed graft function, acute rejection, diabetes mellitus, serious infections or even thrombotic microangiopathy [7,8,9], the precise initial dose tailoring is especially important to achieve the first post-transplant tacrolimus trough level within the therapeutic range, i.e., 5–15 ng/mL [10].

To date, several different approaches were proposed in order to optimize the initial tacrolimus dosing, including clinical factors and CYP3A5 genotyping [11,12,13] as well as computerized dose individualization [14], however not all were successfully validated based on the independent cohort [15]. Taking into account that older and overweight recipients are more prone to develop supratherapeutic first tacrolimus blood levels post-transplant [4,5,16], one could expect that the baseline proportion of fat and lean mass of the recipient may play a role in the post-transplant tacrolimus metabolism [17] and therefore may be another parameter to take into account in the sophisticated process of pre-transplant tacrolimus initial dose calculation. Interestingly, Han et al. reported higher tacrolimus blood trough and 4-h-post-dose levels in stable kidney transplant recipients with the fat mass above median [18]. On the other hand, the frequently observed overhydration in kidney transplant candidates could make the calculation of proper initial tacrolimus dose based on body weight even more difficult. Taking all above evidence together, we hypothesized that some parameters of recipient’s body composition, describing the body water compartments and the proportion of fat and lean mass, would be of value for the optimization of initial tacrolimus dosing. Notably, we did not find such an analysis in the current literature.

Thus, the aim of our prospective study was to investigate the relationship between several recipient’s body composition parameters acquired by bioimpedance analysis (BIA) and the tacrolimus metabolism rate in the first days after kidney transplantation.

## 2. Materials and Methods

### 2.1. Study Group

**Figure 1:**
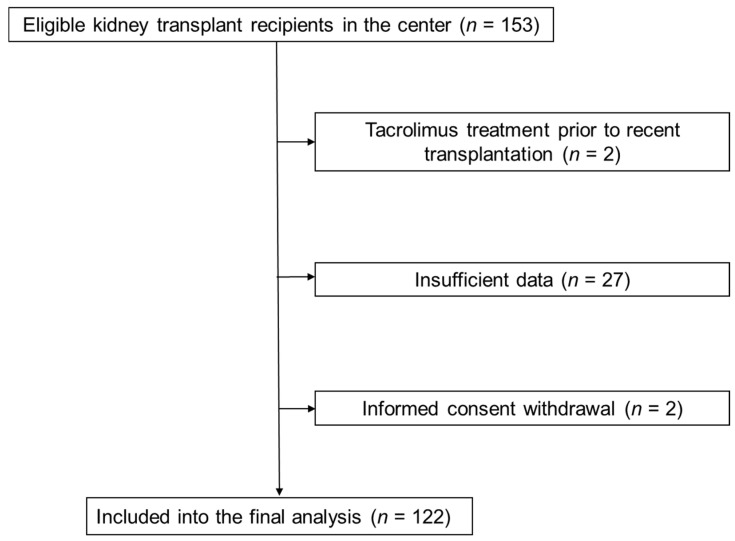
Study flow chart.

We prospectively analyzed all 153 consecutive patients who received their kidney transplant in our center between August 2019 and June 2021. After the exclusion of patients using tacrolimus prior to the most recent transplantation, those with insufficient data and those who withdraw consent to participate, 122 subjects were included in the final analysis (Figure 1). The study was conducted in concordance with the protocol of Helsinki. The Institutional Review Board accepted the study protocol (No KNW/0022/KB1/81/18) and all participants gave their written informed consent.

In each patient, the bioimpedance analysis of body composition was performed immediately prior to kidney transplantation procedure. The first tacrolimus blood trough level was determined within first day’s post-transplantation.

### 2.2. Pre-Transplant BIA

The body composition analysis was performed within few hours before transplantation procedure using BIA device (InBody 770, InBody Japan Inc., Tokyo, Japan) with a multifrequency analyzer (1, 5, 50, 500 and 1000 kHz). All measurements were performed according to the manufacturer’s instructions. Patients refrained from eating for minimum 5 h. During examination, patients stepped barefoot on the footplate containing separate foot electrodes and additionally held the right- and left-hand electrodes. Based on the measurements and BIA software program equations, intracellular water (ICW), extracellular water (ECW), total body water (TBW), ECW/TBW ratio, phase angle, visceral fat area (expressed in cm^2^), lean body mass (LBM) and lean body mass index (LBMI, expressed in kg/m^2^) were calculated. Additionally, the proportion of LBM as a percentage of total body mass was also calculated and analyzed. We also performed the segmental lean analysis in 5 different body sectors: both arms, trunk and both legs.

### 2.3. Immunosuppression Protocol and Tacrolimus C/D Ratio Calculation

All patients received routine immunosuppression regimen, consisted of twice daily tacrolimus (Prograf^®^, Astellas Pharma, Inc., Tokyo, Japan), mycophenolate mofetil and steroids, with an induction therapy using basiliximab or rabbit antithymocyte globulin (rATG). First doses of tacrolimus and mycophenolate were given pre-operatively. Mycophenolate mofetil was started from 750 mg BID. Steroids were given intravenously during operation (500 mg), then 125 mg i.v. the next day and subsequently 20 mg of oral prednisolone daily. Patient receiving rATG was given 125 mg of methylprednisolone instead of prednisolone before each dose.

According to the center protocol, the tacrolimus dose was prescribed based on patients’ body weight. In order to avoid tacrolimus levels exceeding 15 ng/mL, the initial tacrolimus dose was decreased (by 32.9 % in an analyzed group) in patients older than 55 years, with BMI >25 kg/m^2^ and those with the occurrence of anti-HCV antibodies. As patients treated with rATG induction routinely receive antifungal prophylaxis with 100 mg of fluconazole, those subjects were also prescribed a lower tacrolimus dose (usually 3 mg/day—for subjects with body weight below 60 kg—or 4 mg/day for other patients). Based on the first tacrolimus blood trough level measurement, the tacrolimus C/D ratio was calculated.

Delayed graft function (DGF) was defined as a need for dialysis therapy in the first post-transplant week. Acute rejection (AR) was diagnosed based on the results of protocol biopsy performed at a median 8th post-transplant day.

### 2.4. Statistical Analysis

Statistical analyses were performed using Statistica 13.3 PL for Windows (Tibco Inc., Palo Alto, CA, USA) and MedCalc v19.2.1 (MedCalc Software, Mariakerke, Belgium). Values are presented as means with 95% confidence interval, medians with interquartile ranges or frequencies. The main study comparison was performed between groups of patients allocated based on the initially calculated tacrolimus C/D ratio, using the Student t-test (for quantitative variables) or the χ^2^ test (for qualitative variables). Variables with skewed distribution were compared using the Mann-Whitney U test. The second analysis compared patients with LBMI equal and above or lower than median value, using similar tests. Stepwise multiple regression analysis was performed for the tacrolimus C/D ratio as a dependent variable and age, BMI, the amount of residual diuresis, and LBMI as potential independent variables. For all analyses, a *p* value below 0.05 was considered statistically significant.

## 3. Results

### 3.1. Study Group Characteristics

There were 122 kidney transplant recipients recruited into this study. Based on the initial tacrolimus C/D ratio, study participants were divided into two groups, equal and above or below a median value. The baseline characteristics of study groups is given in Table 1. Patients with slower tacrolimus metabolism (C/D ratio ≥ 1.48) were significantly older, more frequently treated with rATG induction as compared with fast metabolizers. Consistently, there was a significant correlation between age and tacrolimus C/D ratio (r = 0.278; *p* < 0.01). There was a tendency to more frequent re-transplants and lower HLA class II mismatch in the fast metabolizers group (Table 1).

There was no difference between the groups in the time from transplantation to the day of the first tacrolimus blood trough level measurement (median 2 (2–3) vs. 2 (2–3) days; *p* = 0.1). As expected, despite the substantially lower initial tacrolimus dosing, slow metabolizers presented significantly higher tacrolimus level and almost 4-fold more subjects reached the supratherapeutic drug level (Table 1). Nevertheless, after the subsequent dose adjustments, the tacrolimus trough levels in both study groups were similar thereafter until the discharge from the hospital (2nd post-transplant week: 9.5 (6.9–10.6) vs. 8.2 (6.8–9.6) ng/mL; *p* = 0.19, 3rd week: 8.4 (6.6–11.4) vs. 9.1 (7.1–10.8) ng/mL; *p* = 0.88, at discharge: 9.4 (7.2–10.9) vs. 8.7 (7.6–9.7) ng/mL; *p* = 0.33, respectively).

DGF was more frequently observed in the slow metabolizers group resulting in a tendency to longer median post-transplant hospital stay (17 (13–25) vs. 14 (13–19) in fast metabolizers, respectively; *p* = 0.07). However, the median serum creatinine concentrations during the hospital stay (3rd post-transplant day: 5.0 (2.8–8.1) vs. 3.5 (2.2–7.9) mg/dL; *p* = 0.29, 7th day: 2.3 (1.3–5.8) vs. 1.9 (1.2–5.5) mg/dL; *p* = 0.33, respectively) and at discharge (1.5 (1.1–2.1) vs. 1.5 (1.1–1.8) mg/dL; *p* = 0.68) were similar. Of note, the frequency of early AR episodes was also similar in both groups (Table 1).

### 3.2. Body Composition Parameters in Slow and Fast Tacrolimus Metabolizers

The BIA parameters describing the pre-transplant hydration status were also comparable between study groups, including the detailed lean mass analysis in 5 individual segments of the body. In line, the median post-transplant weight loss during the hospital stay was similar in both groups (3.1 (2.2–4.1) vs. 3.2 (2.3–4.1) kg in slow and fast metabolism groups, respectively; *p* = 0.90), with comparable values of systolic and diastolic blood pressure measured at 7th post-operative day 9SBP: 135 (130–150) vs. 140 (130–160); *p* = 0.18, DBP: 80 (80–90) vs. 85 (80–90); *p* = 0.55) and at discharge from the hospital (SBP: 130 (120–140) vs. 130 (125–140); *p* = 0.38, DBP: 80 (75–90) vs. 80 (78–85); *p* = 0.49).

Interestingly, we found a significant positive association between the post-transplant weight loss and the amount of pre-transplant residual diuresis (r = 0.304; *p* = 0.01). On the other hand, we also noted a weak reverse association between the residual diuresis and tacrolimus C/D ratio (r = −0.186; *p* < 0.05).

There were no significant differences between both analyzed groups in BIA parameters which reflect the proportion of fat and lean body mass (phase angle, visceral fat area, LBM, LBMI and the proportion of LBM as a percentage of total body mass) (Table 2). In the whole study group, BMI correlated the most with visceral fat area (r = 0.772; *p* < 0.001) and LBMI (r = 0.562; *p* < 001), whereas the correlations with LBM (r = 0.389; *p* < 0.001) and phase angle (r = 0.249; *p* < 0.01) were less pronounced. Of note, we found a weak reverse correlation between LBMI and tacrolimus C/D ratio (r = 0.181; *p* < 0.05). None of the remaining above analyzed parameters correlated significantly with tacrolimus C/D ratio.

### 3.3. Body Composition Parameters in Groups Depending on the LBMI

In additional analysis, in which all study patients were assigned into two groups based on the value of BIA-derived LBMI equal and above or lower than median value, both groups were comparable in recipient age (49.0 (45.8–52.2) vs. 47.4 (44.1–50.8) years, respectively; *p* = 0.50), but the subjects with LBMI ≥ 18.7 kg/m^2^ had significantly greater BMI (27.4 (26.5–28.4) vs. 23.7 (22.6–24.8) kg/m^2^; *p* < 0.001) and residual diuresis (median 600 (250–1500) vs. 175 (0–1000) mL; *p* < 0.01) than subjects with LBMI below median value. They also presented significantly greater phase angle (5.4 (4.8–5.9) vs. 4.7 (4.2–5.3); *p* < 0.001), greater percentage of pre-transplant hydration measured in 5 different body compartments and greater median body weight reduction during the first post-transplant hospitalization (5.3 (2.8–6.3) vs. 1.9 (0.5–3.3) kg; *p* < 0.001).

At baseline, there were no differences in dialysis vintage, CIT, ECW/TBW, and visceral fat area between those groups. However, despite similar initial tacrolimus dose per kg of body weight (median 0.13 (0.09–0.15) vs. 0.14 (0.07–0.15) mg/kg/day; *p* = 0.94), the group of patients with LBMI ≥ 18.7 kg/m^2^ had a significantly lower tacrolimus C/D ratio (median 1.39 (0.94–1.79) vs. 1.67 (1.00–2.28), respectively; *p* < 0.05) in comparison with the other group. As a consequence, regardless of the significantly higher initial tacrolimus dosing in subjects with the LBMI ≥ 18.7 kg/m^2^ (median 12.0 (7.0–13.0) vs. 8.0 (4.0–10.0) mg/day; *p* < 0.001), the first tacrolimus blood trough level was similar in both groups (median 11.8 (7.8–18.5) vs. 10.4 (7.4–15.8) ng/mL, respectively; *p* = 0.32). Multiple regression analysis revealed that age (r_partial_ = 0.322; *p* < 0.001) and LBMI (r_partial_ = −0.254; *p* < 0.01) independently influence the tacrolimus C/D ratio.

## 4. Discussion

To our best knowledge, this is the first study which analyze the association between various parameters of body composition and the early post-transplant tacrolimus metabolism in kidney transplant patients. Hereby, we showed the independent association between the lean body mass index, calculated based on the bioimpedance measurement and the first post-transplant C/D ratio. This finding is of potential importance as it may be useful for the more precise tacrolimus dose determination immediately prior to kidney transplantation.

It is worth to noticing that tacrolimus C/D ratio calculated at 3-month or 6-month post-transplant time-point was previously shown to be a risk factor for significantly worse patient survival, worse kidney graft function and survival, higher rejection rate and the development of calcineurin inhibitor nephrotoxicity and BK nephropathy [19,20,21]. Of note, in our study we used tacrolimus C/D ratio calculated in the first day’s post-transplant as a surrogate marker for tacrolimus metabolism. Interestingly, some metabolic differences defined based on C/D ratio were noted between LCP-tacrolimus and immediate-release tacrolimus during the early post-transplant period [22], however in our study all subjects were treated with an immediate-release drug formulation.

Recently, an increasing interest is observed in the literature in the role of body composition-based pharmacokinetic analyses in an effort to reduce severe drug toxicity. Particularly sarcopenia and body composition in cancer is being studied extensively, resulting in the emergence of body composition-tailored drug administration schemes [23]. As it was shown that dose per kilogram of LBM of the carboplatin was a significant predictor of severe hematologic toxicity, taking body composition into account was proposed for dose individualization of chemotherapeutic agents [24]. In line, computed tomography-derived body composition parameters were correlated with toxicity, dose reduction and termination of the treatment in patients with diffuse lymphoma receiving immunochemotherapy [25]. Moreover, based on cancer research, body composition-based dosing regimens were also proposed for quinolones [26] and anti-tumor necrosis factor medications [27].

In our transplant center, based on clinical observations and previous reports, we started to reduce the initial tacrolimus dose from 0.2 to 0.1–0.15 mg/kg/day in older and overweight/obese patients since 2011. However, as we summarized our experience, we found that such a policy did not decrease the percentage of subjects with supratherapeutic first tacrolimus level substantially [28]. The present study protocol was designed to find potential parameters, which may be obtained prior to transplantation procedure and may help in the optimization of the initial tacrolimus dose. Finally, we confirmed that the bioimpedance body composition analysis of the potential kidney transplant candidate might be useful in such a dose tailoring, as the LBMI was found to be associated with the first post-transplant tacrolimus level independently of age and BMI. In line, of all anthropometric variables tested, a stepwise multiple regression analysis revealed that LBM was the only determinant of antipyrine clearance in apparently healthy subjects [29]. Moreover, LBM was found to best describe the average reported relationship between drug clearance and TBW in literature meta-analysis [30]. Interestingly, in chronic dialysis patients treated with vancomycin, the drug volume of distribution correlated best with LBM [31]. It was also shown in peritoneal dialysis patients that bioimpedance analysis can be used to estimate TBW and LBM with a correlation coefficient of 0.87 and 0.93, when using Deurenberg’s formula [32].

Among the study limitations it should be noticed that the bioimpedance measurements were performed around the clock, when the potential recipients of a kidney from deceased donors were arriving to the transplant center. However, they abstained from eating for a minimum of 5–6 h prior to the examination, as they were aware of the planned transplantation and the pre-transplant dialysis session was performed. Another limitation is the lack of CYP3A5 genotyping in our patients. However, Polish Caucasian population is rather homogenic, with the vast majority of subjects (approximately 90%) being slow calcineurin inhibitor metabolizers [33]. Moreover, to date, CYP3A5 genotyping is neither recommended nor routinely performed prior to transplantation; thus, it cannot be used for initial tacrolimus dose calculation in daily clinical practice. Furthermore, according to some reports, the optimization of initial tacrolimus dose using pharmacogenetics testing was poorly predictive of tacrolimus clearance and did not improve clinical outcomes [15,34,35].

## 5. Conclusions

To conclude, in our present study we found the independent influence of LBMI on the very early tacrolimus C/D ratio post kidney transplantation. Thus, one could expect that based on this immediately assessed, non-invasive body composition parameter it will be possible to more precisely adjust the initial tacrolimus dose in order to minimize its potential toxicity. However, the clinical utility of this novel covariate for the optimization of tacrolimus dosing is still to be proven and needs the prospective validation in the randomized study.

## Figures and Tables

**Table 1:** Baseline characteristics of study subgroups based on the median value of initial tacrolimus C/D ratio.

Parameter	Tacrolimus C/D Ratio		*p*
	Slow Metabolizers ≥1.48 *n* = 61	Fast Metabolizers <1.48 *n* = 61	
Patient			
Age (years)	51.6 (48.5–54.7)	44.7 (41.6–47.9)	<0.01
Sex (M/F)	40/21	36/25	0.46
BMI (kg/m^2^)	25.6 (24.5–26.8)	25.7 (24.6–26.8)	0.95
Dialysis vintage (months) *	34 (25–55)	31 (20–44)	0.29
Residual diuresis (mL) *	300 (0–1000)	500 (100–1500)	0.35
Transplant procedure			
Retransplant (*n*, %)	4 (6.6)	10 (16.4)	0.09
HLA class I mismatch *	2 (2–3)	2 (2–3)	0.48
HLA class II mismatch *	1 (0–1)	1 (0–1)	0.09
CIT (h)	18.7 (16.9–20.4)	17.8 (16.2–19.4)	0.46
Induction therapy			
IL-2RB (n, %)	29 (47.5)	46 (75.4)	<0.01
ATG (n, %)	32 (52.3)	15 (24.6)	
DGF (*n*, %)	19 (31.1)	9 (14.8)	<0.05
Early acute rejection (*n*, %)	5 (8.2)	5 (8.2)	1.0
Tacrolimus dosing and metabolism			
Tacrolimus dose (mg/d) *	7.0 (4.0–12.0)	11.0 (8.0–13.0)	<0.001
Tacrolimus dose per kg (mg/kg) *	0.11 (0.06–0.14)	0.14 (0.12–0.16)	<0.001
Initial tacrolimus level (ng/mL) *	15.5 (9.0–21.6)	9.7 (6.4–12.0)	<0.001
Initial tacrolimus level > 15 ng/mL (%)	50.8	13.1	<0.001
Tacrolimus C/D ratio *	2.00 (1.71–2.50)	0.99 (0.74–1.24)	<0.001

Data presented as means with 95% confidence interval, * medians with Q1–Q3 values or frequencies, as appropriate. C/D, concentration-to-dose; BMI, body mass index; HLA, human leukocyte antigen; CIT, cold ischemia time; IL-2RB, interleukin-2 receptor blocker; ATG, antithymocyte globulin; DGF, delayed graft function.

**Table 2:** The comparison of the main results of bioimpedance analysis between both study subgroups.

Parameter	Tacrolimus C/D Ratio		*p*
	Slow Metabolizers ≥1.48 *n* = 61	Fast Metabolizers <1.48 *n* = 61	
Baseline body composition analysis			
Weight (kg)	74.9 (70.5–79.2)	76.1 (72.4–79.7)	0.67
ICW (L)	24.5 (23.1–25.9)	25.5 (24.1–26.9)	0.31
ECW (L)	15.7 (14.8–16.5)	16.2 (15.3–17.0)	0.42
TBW (L)	40.2 (37.9–42.5)	41.7 (39.4–44.0)	0.35
ECW/TBW	0.390 (0.387–0.393)	0.388 (0.385–0.390)	0.27
Phase angle (^o^)	5.0 (4.8–5.3)	5.2 (5.0–5.4)	0.32
Visceral fat area (cm^2^) *	93.6 (59.1–126.3)	88.4 (51.5–129.9)	0.60
LBM (kg)	51.5 (48.6–54.4)	53.5 (50.5–56.4)	0.34
LBM (%)	69.2 (67.0–71.4)	70.5 (67.7–73.3)	0.45
LBMI (kg/m^2^)	18.6 (17.9–19.3)	18.8 (18.2–19.5)	0.62

Data presented as means with 95% confidence interval, * medians with Q1-Q3 values, as appropriate. C/D, concentration-to-dose; ICW, intracellular water; ECW, extracellular water; TBW, total body water; LBM, lean body mass; LBMI, lean body mass index.

## Data Availability

The datasets generated and/or analyzed during the current study are not publicly available due to limitations of ethical approval involving the patient data and anonymity but are available from the corresponding author on reasonable request.
